# Building partnerships in education through a story-tool based intervention: Parental involvement experiences among families with Roma backgrounds

**DOI:** 10.3389/fpsyg.2023.1012568

**Published:** 2023-03-09

**Authors:** Tânia Moreira, Juliana Martins, Cátia Silva, Emilio Berrocal de Luna, Joana Martins, Daniela Moreira, Pedro Rosário

**Affiliations:** ^1^Department of Applied Psychology, University of Minho, Braga, Portugal; ^2^Departement of Research Methods and Diagnostic in Education, University of Granada, Granada, Spain

**Keywords:** acculturation, home- and school-based parental involvement, academic socialization, Roma (Gypsies), story-tool intervention, home-school partnerships, intervention-based research

## Abstract

**Introduction:**

School educators are likely to explain the poor educational trajectories of students with Roma backgrounds related to the lack of parental support and interest in children’s education. Aiming to understand further the patterns of Roma group’s parental involvement in children’s school life and their engagement experiences in school-related activities, the current research set an intervention supported by a culturally sensitive story-tool.

**Method:**

Grounded in the intervention-based research framework, 12 participants (i.e., mothers) from different Portuguese Roma groups participated in this study. Data was collected through interviews conducted pre-and postintervention. Eight weekly sessions were delivered in the school context, using a story-tool and hands- on activities to generate culturally significant meanings regarding attitudes, beliefs, and values toward children’s educational trajectories.

**Results:**

Through the lens of acculturation theory, data analysis provided important findings under two overarching topics: patterns of parental involvement in children’s school life and participants’ engagement in the intervention program.

**Discussion:**

Data show the distinct ways Roma parents participate in children’s education and the relevance of mainstream contexts providing an atmosphere likely to build collaborative relationships with parents to overcome barriers to parental involvement.

## 1. Introduction

Over the past 20 years, the European Union has been setting policies in various domains (e.g., health, employment, education) oriented to improve the living conditions and inclusion of Roma^1^ communities. Regarding education, the efforts made have failed to achieve the expected benchmarks in closing gaps between Roma and Non-Roma communities (Brüggemann and Friedman, 2017; Alexiadou, 2019). Despite slight improvements (e.g., the school failure rate among the community decreased from 48.1% in 2017 to 37.5% in 2019), Portuguese data on Roma groups (Direção-Geral de Estatísticas da Educação e Ciência [DGEEC], 2019) show that from the children and youth enrolled in school, more than half (68%) attend elementary school levels, and a residual percentage of students (3%) participate in high school levels. This picture mirrors the international statistics pointing out that two-thirds of people with a Roma background aged 16–24 are not attending training, education, or employment in Europe (European Union Agency for Fundamental Rights, 2018).

The poor education trajectories of Roma groups are likely to be explained by a complex network of individual-, familiar-and institutional-related factors (Ward and Geeraert, 2016; Alexiadou and Norberg, 2017; Dimitrova et al., 2017; Rosário et al., 2017; Sime et al., 2018; Alexiadou, 2019). Parental involvement in the school setting has been highlighted in the literature and educators’ speeches as a solution for the disengagement of children, particularly marginalized ethnic students, such as Roma groups (Bhopal and Myers, 2016; Rosário et al., 2016, 2017; Hamilton, 2017; Wauters et al., 2017; Sime et al., 2018; Schneider and Arnot, 2018). Supported by the crucial role of parental involvement in educational outcomes (e.g., positive attitudes towards school, academic achievement), policymakers and school staff have been displaying efforts to enhance home-school relationships (e.g., literacy classes for parents; Gould, 2017); or interventions to increase the family’s participation in educational decision-making processes and school activities among Roma groups (Flecha and Soler, 2013; Khalfaoui et al., 2020). However, families from ethnic marginalized communities may find it challenging to comply with traditional forms of parental involvement (e.g., home-learning supervision and monitoring; parent-teacher in-person meetings) due to acculturation hassles and setbacks (Clifford and Humphries, 2018). For example, low knowledge of the school environment and poor sociocultural competencies to support their children, lack of awareness of the relevance of parental involvement, perceived discrimination, and lack of perceived parenting competencies and skills were found to affect the quality of Roma parental efforts to support children in education (Reynolds et al., 2015; Hill et al., 2016; Clifford and Humphries, 2018; Day and Dotterer, 2018; Parsons et al., 2018; Sime et al., 2018). To narrow the Roma groups parenting quality gaps and expand forms of parental involvement in children’s education, the current study explores a tailored parental program to expand forms of parental involvement in children’s school trajectories.

### 1.1. Acculturation conditions and outcomes: Parental involvement

For marginalized ethnic groups, such as Roma, school learning and social dynamics provide an essential source of intercultural contact (Berry et al., 2006; Ward and Geeraert, 2016; Makarova et al., 2019; Moreira et al., 2022). The ongoing intercultural communication in school pushes individuals to undergo various personal and cultural changes regarding their behaviors, attitudes, and identities (Berry, 2005). This process is known as acculturation (Berry, 1997). Prior research found that the extent individuals engage in mainstream culture or their heritage culture yields distinct acculturation paths. Berry’s model (Berry, 2005) describes four acculturation strategies integration (participation in both native and mainstream cultures), assimilation (participation in mainstream culture over the native culture), separation (retention of native culture over mainstream participation), and marginalization (rejection of both cultures). Under an ecological lens, family and school are the most influential contexts in children and youth’s cultural socialization processes (Wang and Benner, 2016). However, the cultural distance between Roma and non-Roma culture leads to a discontinuity of socialization processes undergone by families and schools. The family sets the foundation for cultural socialization by transmitting values, norms, and traditions of the heritage culture (Phinney et al., 2001; Hughes et al., 2006; Ward and Geeraert, 2016) while acting as crucial allies to support participation in and adoption of the mainstream culture (Tyler et al., 2008; Moreira et al., 2023). Child’s acculturative orientations and outcomes are related to their families’ cultural orientations and expectations (e.g., maintaining heritage culture or adopting the mainstream culture; Schachner et al., 2016; Moreira et al., 2022). Therefore, parents’ attitudes and efforts towards school are likely to encourage children’s socialization in the mainstream culture (Schachner et al., 2014; Dimitrova et al., 2017) and favor positive acculturation outcomes, such as school engagement and success.

Parents support and participate in their children’s education in various ways. For example, Hill and Tyson (2009) categorize parental endeavors (i.e., beliefs, attitudes, and actions) to support and participate in children’s education within a tridimensional model: home-and school-based involvement and academic socialization. Home-based involvement refers to helping and supporting children in their schoolwork, promoting a learning environment at home (e.g., encouraging reading), and communicating with children about school and standard school behaviors (Hill and Tyson, 2009). School-based involvement includes communication between parents and teachers and active participation in school events (Hill and Tyson, 2009). Finally, academic socialization refers to parents’ direct and indirect messages likely to influence children’s school-related outcomes (e.g., parents’ beliefs on the value and utility of education and educational expectations; Hill and Tyson, 2009; Benner et al., 2016). Each expression of parental involvement contributes differently to children’s school success (Fan and Chen, 2001). For example, parental efforts to communicate positive expectations on the value and utility of education for the future (academic socialization) were found to be more related to children’s school achievement than overt strategies of home and school-based involvement (e.g., homework help or participation in school meetings; Hill and Tyson, 2009; Benner et al., 2016).

The ways and the quality of parental involvement vary across cultures and are influenced by school-related factors (e.g., school norms, values, interracial relationships, and teacher support; Gutentag et al., 2018; Göbel and Preusche, 2019; Kramer et al., 2021) beyond individual factors (e.g., personal experiences, perspectives about parenting, and child-rearing goals; Hinton-Smith et al., 2018; Khalfaoui et al., 2020). For example, the intense assimilationist pressures to endorse mainstream culture and attitudes of prejudice and exclusion perceived by families with Roma backgrounds are likely to diminish their school-based involvement (Phinney et al., 2001; Hughes et al., 2006; Ward et al., 2010).

### 1.2. Parenting programs: Enhancing parental involvement

Parental involvement in children’s school activities is difficult to promote and sustain for families from different cultural backgrounds. In particular, because programs and policies designed to foster the participation of parents from mainstream groups may be ineffective when targeting parents from other backgrounds who lack cultural capital (Park and Holloway, 2013; Williams and Sanchez, 2013; Robinson and Harris, 2014; Moreira et al., 2022, 2023). For example, educational efforts focused on promoting traditional forms of parental involvement (e.g., attending school events and checking homework) may fail to address the livelihood singularities of most parents with Roma backgrounds (e.g., Dolean, 2016; Kramer et al., 2021). The literature lacks comprehensive and culturally appropriate programs to tackle specific needs and create opportunities for parents from marginalized ethnic groups to engage in school (e.g., intentional efforts to develop parents’ tacit knowledge of the inner working of the school system; Baquedano-López et al., 2013; Nata and Cadima, 2019; Kramer et al., 2021; Moreira et al., 2023).

Parents’ participation in parenting programs may help them to expand role constructions, parenting practices, perceived self-efficacy, and social support (e.g., Nata and Cadima, 2019). Evidence of the success of programs and initiatives designed to value parents’ existing knowledge and insights has been provided in the literature. Data show that building upon parents’ existing strengths while responding to their challenges is helpful in influencing parents’ motivations and self-efficacy and, therefore, how they perceive the school outreach efforts (i.e., welcoming versus threatening environment; Green et al., 2007; Mapp et al., 2008; Kim, 2009; Park and Holloway, 2013). As Hoover-Dempsey and Sandler (1997) argue, parental role constructions, self-efficacy beliefs, and perceived school environment are crucial aspects to attend to while sustaining parents’ motivation to become and remain involved in their children’s education.

Despite the plethora of programs designed to enhance parental involvement in student learning among culturally or economically deprived groups, the extent to which parents benefit from those programs may differ across social and economic backgrounds (Almeida et al., 2014). There is a lack of information on the extent these programs are effective in narrowing gaps between people from marginalized and their peers from non-marginalized groups (e.g., gaps in tacit knowledge) and incrementing home-school partnerships (Kim, 2009; Hamlin and Flessa, 2016; Nata and Cadima, 2019; Aguiar et al., 2020; Ferreira and Nunes, 2021). For example, in the particular case of marginalized ethnic families, outreach efforts to increment cultural capital (e.g., providing tacit knowledge of the school’s inner workings) may be crucial to narrowing the parenting quality gaps and maximizing parental involvement in education (Hill and Craft, 2003; Kim, 2009; Nata and Cadima, 2019). Taking all together, families with Roma background were invited to enroll in an intervention program tailored to enable parental involvement. Building upon culturally available forms of parents’ involvement in education, the current study explores how key features and principles embedded in the intervention help tackle barriers to program implementation and parents’ effective involvement.

## 2. The current study

Drawing on existing research supporting the effectiveness of social-cognitive interventions to promote positive attitudes and interpersonal relations (Beelmann and Heinemann, 2014); and, on the lack of systematic evidence on programs directed to improve parental competencies to involve in education among Roma groups, an intervention was purposefully designed and implemented (see Methodology section) integrating key features presumed to impact engagement and outcomes in parental-based programs: (i) target group needs and specificities (e.g., parents’ available forms of involvement, cost–benefit perceptions, parents’ strengths, and resources); (ii) program characteristics (e.g., interactive approaches, using group exercises, story-tool); and (iii) process-related factors (e.g., openness to explore cultural meanings and academic aspirations, actively invite them to take part and provide inputs, positive climate and group cohesion). Together these proximal determinants of enrollment are expected to strengthen personal resources (e.g., perceived utility and competence) needed to expand available forms of parental involvement while detaching from deficit-based approaches and reducing the perceived threat underlying parental programs.

The present study is grounded on the ecological nature of acculturation processes (Ward and Geeraert, 2016) and the tridimensional conceptualization of parental involvement (Hill and Tyson, 2009). The general aims are twofold: [1] to depict parental involvement efforts in children’s education among families from Roma groups before and after participating in a parental program, and [2] to assess parents’ perspectives of their engagement experiences and program-related outcomes.

This study adds to the literature on parenting roles and patterns of involvement in education among parents with a Roma background. Moreover, by exploring families’ experience of enrolling and engaging in the intervention program, this study helps to identify critical features and setbacks in different domains underlying parental engagement in school-related activities. We believe that these experiential insights, albeit preliminary, may shed light on how schools may develop more effective partnerships with parents from Roma groups and improve their children’s school success. Hopefully, the knowledge generated will help policymakers, school practitioners, and stakeholders set policies and make effective decisions to facilitate the positive educational outcomes of Roma communities.

### 2.1. Contextual setting

This study was conducted in two public elementary schools in disadvantaged areas. Both schools are on the outskirts of two cities in the north of Portugal. The reasons for selecting these two schools are twofold: the high number of students from the Roma groups enrolled in each school and the diversity of Roma groups in both schools. Despite being portrayed as a homogenous group, in Portugal, Roma groups distinguish themselves by their origin, race’s purity, cultural traditions and values, economic activities, dialect/language, residence area and living conditions, and openness/closeness to mainstream society. Rooted on these intragroup differences, the living areas of each group are well delimited, and individuals from the Roma groups avoid social and marital relationships between groups (Mendes, 2007; Magano, 2010). The schools targeted in this study include families from three Portuguese Roma subcultures, and the differences highlighted by the interviewees are consistent with previous works (Magano, 2012; Valente, 2014).

One of the groups enrolled is from the inner regions of the country’s northeast border, whose principal economic activity is selling balloons and toys in traditional markets and popular fairs. According to the other Roma subcultures, this group is more permeable to mainstream influences. Marriages with different Roma subgroups are not well accepted, favoring intrafamilial unions. Another group comprised individuals settled in social housing, whose principal economic activity is selling garments in fairs (e.g., clothing and footwear). This group, perceived as conservative, lives in a patriarchal structure where gender roles are ascribed, despite women being perceived by the other groups as more active and emancipated. Within this group, marriage occurs later than in other groups. The last group comprised individuals living in tent camps, whose economic activity is selling scrap metal, raising farm animals, and subsistence agriculture. This group has even fewer resources than the other two. Gender imbalance is more prominent, placing women into subservient roles. Early marriages and inbreeding are common in this group. Given their cultural specificities, particularly regarding the resources available and the openness to contact and participate in mainstream systems, parents are expected to hold different personal resources and face other challenges while participating in their children’s education.

Moreover, it is also hypothesized that cultural differences can shape the extent to which parents engage in the intervention. For example, groups living in tents may face additional challenges (e.g., logistical and language barriers) to participate in their children’s education. On the other hand, the group settled in social housing in urban areas are potentially more exposed to perceived discrimination in mainstream settings (e.g., compulsory parenting programs), being more resistant to engage in the intervention.

## 3. Methodology

The current study is grounded on the intervention-based research framework, widely used in education as a powerful tool to conceptualize, assess, and intervene in the participants’ lives (Hu et al., 2021). This design was used to understand the patterns of parental involvement in children’s school life among Roma families while promoting opportunities to support and expand parenting roles and their forms of involvement in children’s school life. The intervention protocol was submitted and approved by the Ministry of Education and the Scientific Ethics Committee of the University.

### 3.1. Story-tool based intervention

A collaborative intervention using a story-tool was developed with two groups of parents with Roma’s background.

#### 3.1.1. The story-tool

Storytelling is a powerful educational tool that provides opportunities to generate culturally significant meanings regarding attitudes, beliefs, and values (e.g., Rosário et al., 2010; Greenfields et al., 2015; Rosário et al., 2016). Listeners can identify themselves with settings and characters, which may prompt reflection experiences and encourage behavior change (Palacios et al., 2015; Lee et al., 2016). Moreover, prior research has suggested that storytelling is most effective in cultures with a strong oral tradition, such as Roma groups (see Haigh and Hardy, 2011; Visconti, 2016). Grounded on these reasons, a narrative was purposefully created with meaningful elements related to cultural traditions and values and the roles played by the parents on younger individuals’ life trajectories. The initial version of the story-tool was discussed with researchers and social work assistants with experience working with Roma communities and with community members (cultural mediators) to reduce unconscious bias and calibrate materials’ cultural relevancy and literacy level. The suggestions made were incorporated into the final version. The narrative comprises a frame story (Matryoshka Stories), where the main report subsumes a set of shorter stories (see an overview in Supplementary Table 1). The current history depicts the tale of Musca, a little and thin arctic swallow with approximately 120gr, who, following her family tradition, flew from the Arctic to the Antarctic. Musca ran from the harsh cold of the arctic to meet summer on the other side of the Earth. Along this long journey, Musca stopped resting on an old tree branch somewhere in Africa. While resting, Musca meets local birds, tells stories from her trip around the Earth, and learns games and local stories from her new friends. A repertoire of self-regulation strategies is embedded in the narrative (e.g., goal setting), which is explored using declarative, procedural, and conditional knowledge (Núñez et al., 2013; Supplementary Table 2). Participants are invited to look inside their own lives and prompted to discuss and build their cultural meanings while guided to reflect, for example, on the merits of distinct education contexts, educators’ roles, life expectations, and cultural challenges. The discussion of the narrative allows participants to reflect on the story plot while reasoning on their life circumstances and challenges (e.g., discuss how the little and fragile arctic swallow managed to overcome the obstacles and difficulties found along the way and attain her goal; or how the family history and tradition shaped Musca’s pathways). This experiential closeness and cultural sensitivity are likely to foster parents’ engagement with the discussion and enhance the development of positive attitudes toward the strategic contents introduced by the narrative (Rosário et al., 2016).

### 3.2. Procedure

A meeting was organized in both schools to inform parents about the program. The aim was to connect with the community and familiarize researchers with their cultural aspects while establishing trusting relationships. One group was composed of two Roma communities, and the other group was more homogenous regarding their Roma origin (i.e., comprising of Roma parents living in tents). Eight 90-min weekly sessions took place for each group in schools, using rooms prepared for small-group discussions. No rewards or payments were offered for participation. Two implementers (both from non-Roma groups) with a background in educational psychology delivered the program in both groups (with 6 and 7 mothers, respectively). The leading implementer had previous experience working and researching with Roma groups and extensive training in multicultural approaches and practices. The second implementer received training on Roma culture references and multicultural approaches for the current research. This training was delivered by the senior researcher and Roma community members. The sessions were offered in Portuguese (the common language for all the groups) and followed an instructional sequence: (i) the sessions started from the first session onwards with an initial recap of the story and the “take-home message” from the previous session; (ii) afterward, implementers read aloud a short chapter of the narrative related to the session’s overarching goal. Participants’ knowledge of the vocabulary used in the story-tool was monitored, and sentences were often translated into everyday language to ensure understanding. Also, participants were encouraged to retell the story in their own words. (iii) The discussion on the session’s topic (e.g., building a shared understanding of the role of formal education in their child’s life, reflecting on parental roles and life expectations and their influence on children’s positive pathways) was prompted by questions. The discussion began with descriptive narrative-grounded questions (e.g., Who are the story’s characters? What is this chapter about? How did the characters overcome their barriers/difficulties?). Afterward, questions were transferred to real-life related inquiries (e.g., In what circumstances do we face similar problems or conflicts in our life? What can we learn from this story?). Participants were purposefully guided to transfer the ideas discussed (e.g., lessons learned) to the educational trajectories of their children. (iv) Sessions also included hands-on activities (e.g., kneading the plasticine into forming a shape, making origami) to help participants further understand and consolidate reflections made while discussing topics. These activities were discussed using the reasoning line of inquiry to articulate and connect knowledge learned with actions to follow and real-life events. Finally, (v) sessions ended with a summary and a brief ‘take-home message’. The questioning flow was responsive to the participants’ answers (discussion flow is depicted in Supplementary Figure 1).

To ensure the trustworthiness of the intervention, procedures were adopted as follows: sessions were fully scripted in a detailed protocol of the intervention (e.g., program purposes, topics to cover, strategies to check participants’ understanding, session activities, and a set of crucial questions to introduce and explore the topic), conveyed to the implementers. These researchers received a 2-day training for delivering this intervention. The training covered the program’s theoretical framework, objectives, procedures, and information on the Roma culture and groups targeted. Moreover, weekly debriefing meetings with a senior researcher were held to review the intervention script and to monitor protocol adherence; additionally, intervention sessions were audio-recorded and checked against the protocol by two researchers. The overall fidelity of delivery (fidelity to structure and process; Mowbray et al., 2003) ranged from 85 to 95% across sessions for both groups.

#### 3.2.1. Data collection

A purposive sampling strategy was used to recruit participants to ensure diversity within the target population (Robinson, 2014). The project was presented to school directors and social work assistants; afterward, a meeting with parents (mothers and fathers or other adult caregivers) from the Roma community was held to invite them while providing all the program-related information. Families selected which members enrolled in the program. Like in many cultural groups, mothers with Roma backgrounds assume a crucial role in children’s school trajectories. Thus, despite the openness shown to fathers to participate, the groups enrolled in the current study were mainly comprised of mothers with children attending school (from the last years of elementary school onwards). Individual informed consent was provided to families who agreed to participate. The participants were invited as full partners rather than passive participants; for example, prescriptions on how to educate their children were not discussed in the sessions. In other words, the intervention design aimed to help participants reflect on their meanings, roles, and actions and anticipate the implications on children’s education trajectories while encouraging their agency and personal control. Moreover, reflecting on their resources, parents were encouraged to build reachable strategies to participate in children’s school life.

Two of the authors collected data through semi-structured interviews in two waves. The first data wave was collected before the beginning of the intervention. This interview was initiated with general information about the program to overcome mistrust and cultural and linguistic barriers and help establish relationships with the participants (Lyberg et al., 2014). The interview included open questions focused on participants’ beliefs on the value of education, academic expectations, parenting roles, and practices regarding their children’s education. Follow-up questions about their expectations regarding the parenting program and feedback on the interviews were also included in the interview script. The second data collection interview added a set of questions about participants’ experiences while enrolling and engaging in the intervention program. The questions’ wording was adjusted to overcome language barriers (i.e., some participants struggle to use proficiently the Portuguese language), misunderstandings, and expression gaps as needed. Interviews lasted between 30 and 45 min and were audio recorded and transcribed verbatim, ensuring anonymity and confidentiality.

#### 3.2.2. Sample

School directors and social work assistants facilitated contact with the community. Finally, 17 mothers agreed to participate in the program. Five participants missed several sessions and were not interviewed in the second data collection wave. Reasons were related to sickness and medical appointments (e.g., cancer diagnosis), problems at home (e.g., the apartment burned down, family resettlement), and overlapping household chores. Finally, data analysis drew on 13 participants who attended seven (*n* = 3) or eight (*n* = 10) program sessions; these women were interviewed in the two data collection waves (24 interviews, see Table 1). Participants ranged from 27 to 51 years old (*M* = 36.4; *SD* = 7.2).

**Table 1:** Descriptive information about the cases.

	Age	Academic qualifications	Literacy	Community of origin
Toya	27	*	Illiterate	A
Charani	31	6^th^ grade	Read and write	C
Ashila	31	4^th^ grade	Struggle to read and write	C
Samara	32	4^th^ grade	Read and write	A
Eldra	32	*	Illiterate	A
Dorelia	33	3^rd^ grade	Struggle to read and write	B
Masilda	34	*	Illiterate	C
Deloreni	38	1^st^ grade	Illiterate	C
Mary	42	**	Illiterate	C
Analetta	43	*	Illiterate	C
Everilda	44	6^th^ grade*	Struggle to read and write	C
Ostelinda	50	*	Illiterate	B
Selesia	51	3^rd^ grade*	Struggle to read and write	B

A – Living on the northeast border of the country and selling balloons and toys in popular markets and fairs; B – Living in urban areas in social housing, whose principal economic activity is selling garments in fairs; C – Living in rural areas in tent camps whose economic activity is related to selling scrap metal, animals, and subsistence agriculture.

* No school attendance.

** Attended an adult literacy course.

Regarding education, half of the sample (50%) had never attended school during childhood (women aged between 27 and 43 years old). From those, some attended adulthood introductory literacy courses to develop reading and writing skills. Most participants who attended school as a child completed elementary school (67%).

#### 3.2.3. Data analysis

Data analysis focused on (1) patterns of parental involvement in children’s schooling and (2) participants’ experience of enrolling and engaging in the program. Following the recursive process by Braun and Clarke (2012), a thematic content analysis was performed, helped by the NVivo software. Interviews were coded using a “hybrid approach” (e.g., Swain, 2018). First, data was explored in an inductive way to identify emergent codes. A deductive logic was added to group codes into previously identified domains (parental expectations, home communication, parents-school communication, parental practices and participation in school, barriers, benefits, and challenges). Analyses were conducted between and within cases, using a constant comparative method to identify potential relations. Relationships and repeated patterns of meaning were identified within the women’s narrative accounts and across participants using software tools to search through the datasets (i.e., queries, cluster analysis, graphical maps). Subsequently, codes assigned to specific domains were grouped into themes previously found in the literature. Demographic data related to parents (e.g., education level; Roma group origin) and children (e.g., gender, age) were considered in the data analysis to search for patterns in data. The scoring scheme proposed by Rodgers and Cooper’s (2006) for qualitative thematic analysis was used to aid clarity in the reporting process. The frequency of participants coded for each outlined subtheme was reported as follows: ‘All’ = 100% (*n* = 13), ‘nearly all’ = 100%−2 participants (*n* = 11), ‘most’ = 50% + 1 to 100%-2 (*n* = 7 to 10), ‘around half’ = 50% + 1 participants (*n* = 6), ‘some’ = 3 to 50% + 1 participants (*n* = 3 to 5), ‘a couple’ = 2 participants, and ‘one’ = one participant. To enhance the trustworthiness of the current findings (Lincoln and Guba, 1985), a random selection of 10 interviews were coded by two researchers independently, and the consensus was reached through discussion. According to Landis and Koch, the kappa coefficient ranged from 0.87 to 0.98, which is considered very good (Landis and Koch, 1977). Verbatim supporting quotes were included to illustrate themes and subthemes, to provide a detailed description of participants’ representations and experiences, and to add validity to the results (Smith, 2017). Pseudonyms were used to identify participants.

## 4. Results

**Figure 1:**
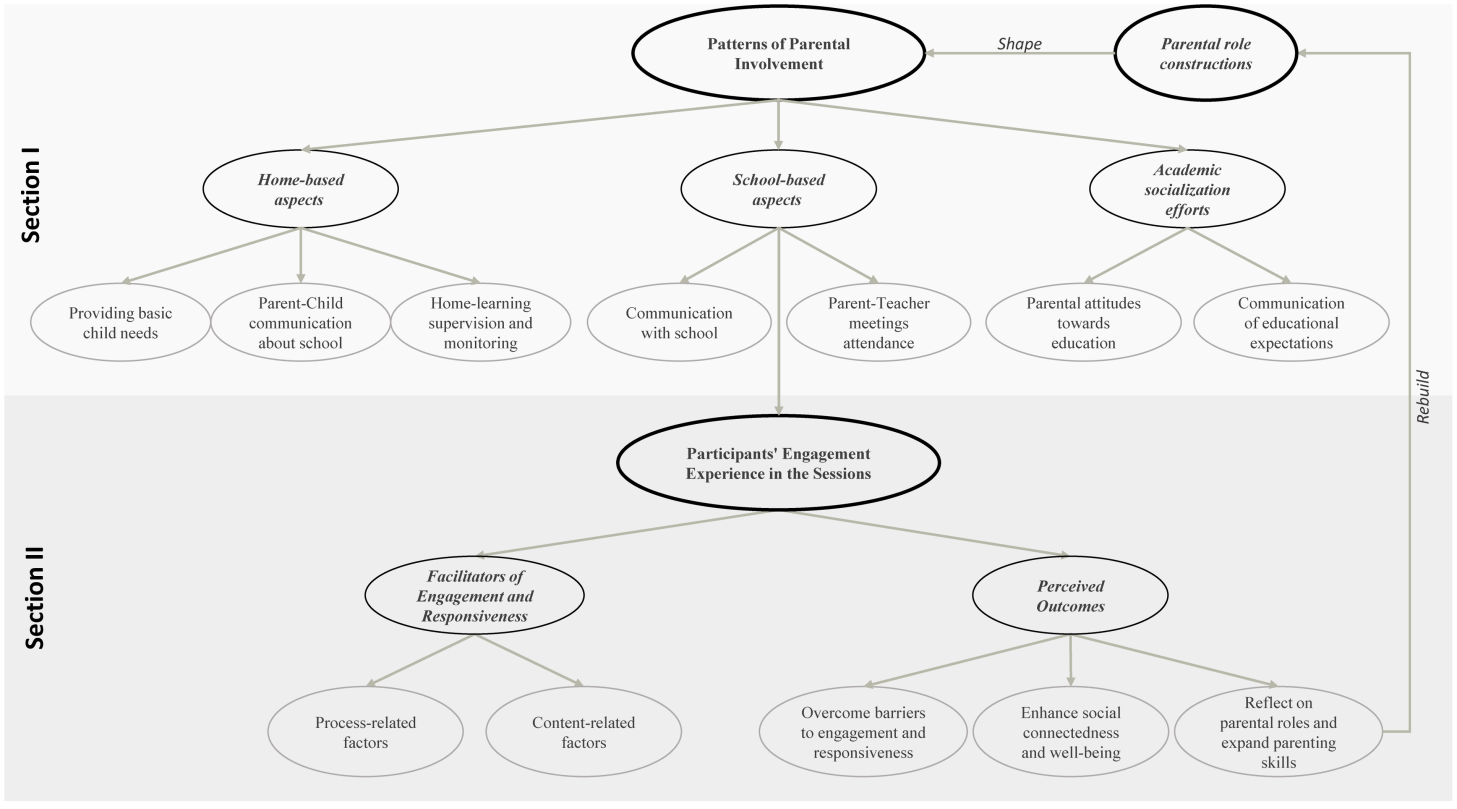
Visual map of the themes, subthemes, and emerging relationships developed through thematic analysis.

Results were organized in two sections: (1) patterns of parental involvement in children’s school life and (2) participants’ engagement experience in the intervention program. A visual depiction of results containing key themes, subthemes, and relationships among them is presented in Figure 1.

### 4.1. Section I. Patterns of parental involvement in children’ school life

Participants were asked about their perceived parenting roles in supporting children’s school trajectories. When comparing reports across both data collection moments, no meaningful differences were found regarding the practices reported by parents on their involvement in children’s school life. Moreover, differences did not emerge in data when accounting for participants’ literacy or children’s gender. However, when layering results by the Roma group living conditions (living in urban areas versus living in tents), slight differences emerged within themes. The emerging differences in participants’ reports were pointed out within each theme.

Participants reported behaviors and attitudes to support and take part in their children’s school life fell into three major themes: (1) home-based efforts, (2) school-based efforts, and (3) academic socialization efforts. The efforts displayed by parents seem to be triggered by the institutional protocols and demands (e.g., school, social security). These influences likely shape parenting role constructions and, therefore, the extent to which parents participate in children’s education.

#### 4.1.1. Theme 1. Home-based efforts

This theme includes participants’ attitudes and practices geared to contribute to their children’s educational outcomes (Hill and Tyson, 2009; Benner et al., 2016; Jeynes, 2018). It covers three subthemes: *providing basic child needs*, *parent–child communication about the school*, and *home-learning supervision and monitoring*.

*Providing basic child needs.* Participants’ parenting role representations translate to educational practices promoting autonomy and physical and economic independence from an early age. For example, all participants reported that children are autonomous regarding the time to wake up and prepare themselves to be at school on time.

“I don’t wake up before them. They are already used to waking up alone and getting ready for school. I tell them, is everything here [in the kitchen] I will not prepare your breakfast. My oldest daughter is used to waking up, getting ready for school, and helping their brothers. They prepare the coffee, drink it, and then go to school. It is more or less like this.” (Samara, read and write, urban-living community)

“When it is 7 o’clock, half past six, he is already woken up to go to school. Even when he is home, he has this routine. He is like that, puts everything in his backpack, dresses up, and prepares everything. Even the breakfast.” (Charani, read and write, rural-living community)

However, when layering results by living community, rural-living participants seemed to express more concern about supervising children in meeting the school schedule (e.g., making sure children wake up and prepare breakfast) and ensuring that children get safely to school. These participants emphasized the latter as essential duties of their role in children’s education.

“He wakes up alone, and he does everything alone. Wash teeth, put the books in the backpack with the things to the school. He eats breakfast, takes the bus, and goes. But I wake up to check if he got the bus. Sometimes when is late, I take him to school. And I also go there to take him back home when he has classes late in the afternoon. I am afraid because there is a street without a crossing area” (Masilda, illiterate, rural living community)

“At six [in the morning] I prepare lunch [breakfast] to be close to my daughter, and she has [enough] time to eat. She is afraid of going alone [to the bus stop], and I walk to the bus with her.” (Analetta, illiterate, rural living community)

*Parent–child communication about school*. Despite not taking the initiative, most participants in our sample referred to talking with their children about their behaviors and activities in school. However, it is worth noting that, in general, participants lack information about their children’s preferences, school tasks, difficulties in progress, or school grade records.

“She never talked about that [preferences and difficulties to solve school tasks]” (Dorelia, struggle to read and write, urban-living community)

“He doesn’t talk, never say a word about the school. He never says what he likes at school or if everything was okay in the classes. He is very quiet, like his dead grandfather.” (Everilda, illiterate, rural living community)

*Home-learning supervision and monitoring*. Nearly all participants noted that they do not have the literacy skills to support their children while completing home-learning activities or checking whether they have completed their homework (note that most of the participants struggle to read or write). To answer the school staff pressures, some participants in both groups referred to display thwarted efforts to monitor children’s homework and study time, such as asking their children whether they had homework assigned or checking their notebooks to learn if they did homework. However, school assignments are commonly expected to be completed on after-school community-based projects. Thus, nearly all participants in our sample mentioned that they do not set time at home for their children’s homework and study.

“When he arrives home, I ask if he has homework.. He tells me that he did homework and I ask him to show me the notebook to see if it is true … but I don’t know how to read.” (Eldra, illiterate, urban-living community)

“After school, she goes to the Red Cross [to do the homework and study]. When she arrives home, eats, and uses the mobile phone all the time.” (Analetta, illiterate, rural-living community)

#### 4.1.2. Theme 2. School-based efforts

School-based aspects include participants’ efforts to communicate with and participate in school-promoted activities and parent-teacher meetings (Hill and Tyson, 2009; Benner et al., 2016; Jeynes, 2018). Nearly all participants reported taking a passive role in the communication flow with the school, responding to requests made by the school (e.g., signing authorizations and attending parent-teacher meetings). Participants reported behaviors and practices fall under the two outlined subthemes: *communication with the school* and *teacher-parent meetings attendance.*

*Communication with the school.* All participants assumed a passive role while relying on the school staff to communicate by phone to update them about their children’s misbehaviors in school (e.g., class non-attendance) and material or homework shortages. Almost all the participants stated that they usually communicate with the school’s social work assistants. In contrast, some participants reported starting contact with the teacher representative to address their concerns about their children’s or peers’ behaviors.

“[I know what happens in school] because I am connected. The social assistant calls me: ‘I am talking with Toya? It happened this and this and this. It is like that … And she tells me what he does in school, that I have to control him, and that he will be punished in school.. Like this.” “When he misses a class or has bad grades, the teachers call me to a meeting.” (Toya, illiterate, urban-living community)

“Sometimes, I call the teacher. When he [child] doesn’t want to go to school, I talk with her [teacher] to ask her to talk with him, to give him some advice, and she does it” (Everilda, struggle to read and write, rural-living community)

*Parent-teacher meetings attendance.* Around half of the participants in our sample referred to attending in-person meetings set by teachers. Participants added that meetings’ agenda usually includes discussion on severe problem behaviors, poor grades records, or administrative tasks (e.g., filling in or signing papers).

“When he misbehaves, they [the social work assistant] call me or send a letter to come to school.” (Ashila, struggle to read and write, rural-living community)

“When there is a problem, they call me immediately to come to school. When they cannot handle her anymore, they call me to the school. It is like this, and I have to come. I know everything bad that happens in school” (Dorelia, struggle to read and write, urban-living community)

#### 4.1.3. Theme 3. Academic socialization efforts

Participants also reported engaging in efforts to instill the value of education for children’s future and career plans. This theme covers *parental attitudes towards education* and *communication of educational expectations*.

*Parental attitudes towards education.* Nearly all participants reported focusing their educational efforts on setting conditions to help children attend school. Participants reported conveying educational messages regarding the importance of education and making efforts to socialize children with school norms and values (e.g., respecting teachers and peers and complying with the rules). When children misbehave, nearly all the participants reported a list of punishment strategies (e.g., verbal reprimands) or withdrawal of privileges (e.g., taking the mobile phone away) used to control children’s behaviors in school.

“I think we have to encourage them to go [to school] because it is good for their future. If we support them, they will not quit. I always tell them to behave well and respect the teacher. My children must obey and behave well; that’s what I teach them.” (Samara, read and write, urban-living community)

“The phone distracts her [in class]. Sometimes I keep the phone at home; she can only use it back at home. I do that [to help her be focused on the class].” (Analetta, illiterate, rural-living community)

*Communication of educational expectations.* Nearly all participants aspire for their children to enroll in school beyond elementary grades. However, participants’ statements reflect poor literacy skills and a lack of tacit knowledge of the school system. This led participants to set vague or unrealistic academic expectations for their children. Overall, participants’ expectations regarding education reflect parental socialization emphasis on fostering young children’s personal and economic independence and quality of life, such as getting a driving license and having an unskilled job or enrolling in a funded professional training course.

“So, I don’t know until what age they should study, I have no idea about this. I would like him to study until he can, until … how can I explain?.. until he is 18 years old, to have a better future. Yes, I expect him to study until he's 18. To have at least completed the eighth grade, I don’t know [In Portugal, at this age youth are expected to complete the 12^th^ grade]. (Eldra, illiterate, urban-living community)

“I would like him to stay [in the school] up to 20 years old. At that age, he would be a veterinarian [her child is 14 years old and currently attending the 5^th^ grade. In the Portuguese educational system, completing the 12^th^ grade is mandatory]. I would like that. He could be called by people with animals and earn money. He has animals and needs a veterinarian; as he would be a veterinarian, he would not need to call one.” (Deloreni, illiterate, rural-living community)

It is worth noting that, while layering findings by the group’s living conditions (urban-living versus rural-living communities), the traditional pathways of Roma people emerge as a barrier to higher academic expectations and school progression, mainly in the urban-living participants’ speeches.

“I would like him to be a policeman, but it is not like that for us. I cannot answer that [academic expectations] because we [Roma community] think differently; when we reach the age of majority, then we leave school. But I would like him to be in school [completing high school].” (Toya, illiterate, urban-living community)

### 4.2. Section 2. Participants’ engagement experience in the sessions

Participants were asked about their expectations for and experiences of the sessions. Two themes emerged: *Facilitators of engagement and responsiveness* and *program perceived outcomes.* Participants shared that their participation in the program helped them improve their awareness of parenting roles and strategies to support children in school. Overall, participants reported positive evaluations of their experience in the program sessions. Layering findings by the Roma group’s living conditions (urban-living versus rural-living communities), slight differences emerged within themes. These differences were pointed out within each theme.

#### 4.2.1. Theme 1. Facilitators of engagement and responsiveness

Participants’ positive evaluation was mainly related to the relationships they developed within the group, the program contents, and the instructional methods followed to convey the contents. Participants’ answers fall into the following subthemes: *Process-related* and *Content-related factors.*

*Process-related factors.* All participants in both groups shared that the process (i.e., group-level dynamics providing an open and safe environment and interaction with other participants) impacted how and how they engaged with the sessions.

“[In the sessions] I felt like it was a relief. I shared my happiness of being a mother, I felt comfortable in sharing also some difficulties I have with my children” (Samara, read and write, urban-living community)

“[In the sessions] I enjoy to be with them, socializing, I don’t know, getting to know each other, giving our opinions, these things.” (Masilda, illiterate, rural living community)

In particular, despite the specific barriers identified by the rural-living community (e.g., long-distance they walk to attend the sessions and the overlap with household chores), all participants emphasized the relationships built and the feeling of being listened to as enhancers of their behavior and emotional engagement in sessions.

“And I had to come on foot. Only God knows; to attend, I left everything behind, the children there … I came sweating and tired, and then I had the housework. But I said: ‘they are waiting for me there, and I have to go’. Then I came and distracted myself … This helped me a lot … believe me. Sometimes I was sad at home, but when I came here, we were together, we played and learned good things, and we talked about our children. I really return back home better. Here, I distracted myself” (Deloreni, illiterate, rural-living community)

*Content-related factors.* Participants in both groups noted program contents and delivery strategies as enhancers of their emotional and cognitive engagement. All participants agreed that the program activities (i.e., story-tool plot and hands-on activities) matched their literacy level and reasoning abilities (e.g., the story was read and pictured, and non-common words were explained thoroughly). This helped participants to overcome obstacles related to poor literacy and engage in discussions. Moreover, the non-directive approach provided opportunities to think over the session topics within a safe environment to open up and share their feelings, opinions, and concerns, enhancing their emotional and cognitive engagement.

“The sessions were excellent. I was not expecting it; it was a good surprise [the sessions]. Like a little box full of surprises … If I read the story for myself, it was a normal story, but no, her discussion was completely different; I understood everything, beautiful, surprising (…). It helped me a lot. It helped me to give more value to things [in my life, with my children]. I want more sessions.” (Samara, read and write, urban-living community)

“I liked these sessions very much. We were together; we had fun, we played and learned many good things, it was good. We learned many things from each other. We learned that it's good to talk to the kids, and see how they spent their day, I think it was. Next year we should return.” (Charani, read and write, rural-living community)

For urban-living participants who are often exposed to parental interventions, the approach followed to deliver the content was positively acknowledged in contrast with previous experiences teaching participants how to educate their children.

“People that do not attend or quit these courses [parenting programs] do it because they hear ‘this is to give education to our children, and they think, but we do not give education to our children already? They [Roma people] think, what do they [social educators] know of how we educate our children? They [Roma people] think like this. In my opinion, the others quit these sessions because they thought that would be the same … but not!” (Samara, read and write, urban-living community).

#### 4.2.2. Theme 2. Perceived outcomes

While sharing their initial expectations, nearly all participants looked at the sessions as opportunities to grow as educators and help their children succeed. Moreover, around half of the participants also reported perceiving their participation in the program as an opportunity for closer contact with people from other cultural groups (e.g., other Roma groups and mainstream people). For example, participants living in urban areas stressed the opportunity to interact with other Roma, while participants living in rural areas referred to the opportunity to contact with mainstream individuals. Ultimately, all participants shared positive outcomes from their session’s enrollment. This theme includes qualitative indicators about the perceived program’s impact on participants, their livelihood, and aspects learned across the sessions. Program perceived outcomes fall into three outlined subthemes: *Overcome barriers to engagement and responsiveness, enhance social connectedness and well-being, reflect on parental roles and expand parenting skills.*

*Overcome barriers to engagement and responsiveness.* Overall, participants in both groups identified potential barriers (e.g., logistic barriers and dispositional barriers) to their participation in the sessions. When layering findings by the Roma group living conditions, most of the urban-living participants assumed an initial resistance to enrolling in the sessions related to their previous experiences in ‘how to parent’ interventions. On the other hand, rural-living participants tend to identify logistic barriers related to long-distance walking, which compromises their home-caring tasks. However, in both groups, participants assumed that their disposition shifted along the program (clearly more positive at the end of the program). Furthermore, they elaborated on the efforts displayed to engage in the program and overcome the dispositional and logistic barriers to participation.

“Once we had to attend something like this. I attended one session. It was even here at this school. I was afraid that this could be the same thing. But then I realized that this would be different. I've never seen ladies like you working with people like us, only gypsies … I really enjoyed the experience. I even think, in my opinion, that this is good for people with our ethnicity because we think we are … we have left aside as if we were not important to society. I think this is very good” (Toya, illiterate, urban-living community)

“Yes, sometimes I arrived nervous [in the sessions] because I had to make food for my husband [prepare lunch before leaving the house] because when I arrive home [at the end of the session], I had a little time [to do house chores] … Still, I never missed [a session].” (Everilda, struggle to read and write, rural-living community)

*Enhance social connectedness and well-being.* Overall, participants in both groups mentioned that participating in the program was an opportunity to experience different social experiences, such as socializing with people from other cultural backgrounds. When analyzing patterns in data, almost all rural-living participants described their participation in the sessions as positive for their well-being while providing opportunities to leave their homes, helping to distract from family problems, and improving their mood.

“What I liked the most was being together (…) [these sessions] put Roma and non-Roma to talk.” (Selesia, struggle to read and write, urban-living community)

“These [sessions] helped distract my head [put away the problems and worries]. Was good because I am a woman that never leaves the house, I am always closed in my life. My head was happy here.” (Analetta, illiterate, rural-living community)

*Reflect on parental roles and expand parenting skills.* Nearly all participants in both groups recognized the valuable opportunities provided to reflect on their parenting role representations regarding the support in children’s education. Participants were encouraged to translate session reflections to their personal lives and parenting representations during the session. For example, in one session participants were invited to knead the plasticine into a shape. The purpose of this goal-directed activity was to elicit participants’ reflections on the roles they may play in children’s lives. All participants in both groups described insights into their parenting representations gleaned from the development of this activity. Also, the reflective exercises following the story-tool passages were pointed out as relevant opportunities to promote self-reflection on their parental roles. When comparing data across moments (i.e., pre-to post-test) between groups (urban and rural-living communities), slight differences regarding parental role constructions were noted. In particular, rural-living participants (i.e., those living in tents) expressed that they learned critical ways to help their children grow and develop in the sessions. For example, these participants mentioned having acknowledged the importance of talking with children about their future perspectives and engaging in efforts to monitor their progress in school better. At the end of the program, rural-living participants expressed that more than just supporting their children’s decisions, they wanted to be involved in academic endeavors because that can make a difference in children’s trajectories.

“I liked the story that tells that you need to get up and continue when you fall. For example, when we, for example, want a job, to work doing anything, we have to fight for that. We cannot sit and wait; we must look and fight, fight. We must fight for our children; we want what is good for them.” (Masilda, illiterate, rural living community)

“[I learned that the] Plasticine were our children, and we had to make something with them [like we were doing with the Plasticine]. [I thought that] We need to put efforts and do beautiful things with them to their future.” (Deloreni, illiterate, rural-living community)

## 5. Discussion

The current study aimed to deepen our understanding of parental involvement in children’s school life among families with a Roma background and build evidence likely to help support school efforts and future interventions on this topic.

### 5.1. Section I. Patterns of parental involvement In children’s school life

In line with previous studies targeting ethnic minorities (Grace and Gerdes, 2019; Garcia and Guzman, 2020; Strataki and Petrogiannis, 2020), current findings show that participants’ parenting role representations affect how they engage in children’s school life. For example, assuming a passive role in academic endeavors, participants reported being engaged on tasks and activities before and after school. Thus, focusing their actions on setting the conditions for the learning process (e.g., providing for the child’s basic needs and ensuring attendance and punctuality to school; picking them up at school or the bus station, and supervising the organization of the school backpacks; respectively), participants rely on school educators the responsibility of their children’s learning (e.g., children’s learning difficulties in reading or writing; difficulties in understanding the content of the homework exercises). Parents generally understand the school’s learning activities as confined to school space and time, expecting schools and educators to support children’s instructional needs to progress.

Nevertheless, building on the multidimensional conceptualization of parental involvement proposed by Hill and Tyson (2009), participants’ narratives reflect parents’ efforts and attempts to participate in different activities and practices to support children in education, particularly home-based efforts. For example, consistent with previous findings (Sime et al., 2018; Strataki and Petrogiannis, 2020), participants described efforts to help children understand the utility of education for the future and foster children’s school attendance. In addition, as a response to assimilative school pressures (Schachner et al., 2017), participants also reported efforts to conform to prevailing forms of parental involvement. For example, participants illustrated attempts to monitor their children’s behaviors at home (e.g., check children’s homework) as a response to school demands to adopt traditional forms of parental involvement within the community (e.g., social work phone calls reporting missed assignments). However, as parents mentioned, the efficacy of these efforts is limited because participants lack the basic literacy skills and cultural capital needed to support children in education. For example, despite the participants’ school levels, no differences emerged regarding parental involvement efforts in children’s education. Current evidence supports previous studies (e.g., Valdez et al., 2013; Makarova et al., 2021), stating that parents’ educational level and social capital hamper the efforts to get involved in their children’s education or reduce the efficacy of those efforts. For instance, participants mentioned communicating with children about the utility of education and educational expectations. However, they are likely to set unrealistic academic expectations for children due to their limited understanding of the educational process (e.g., some parents stated that their children could be enrolled in school until 18 years old and complete the 8^th^ grade; then leave school, work with animals in a small farm, and finally act as a veterinarian without further education). Parents limited tacit knowledge and grade-level expectations, along with their poor perceived efficacy on how to be involved in children’s school life, may help explain their tendency to reproduce in their lives the low expectations communicated by educational policies across generations (e.g., Flecha and Soler, 2013; Battaglia and Lebedinski, 2015; Burchardt et al., 2018; Grace and Gerdes, 2019). Supported by this evidence, schools should consider displaying intentional efforts to provide opportunities for students with Roma backgrounds to develop cultural capital and solid academic identities.

Overall, our data are consistent with previous findings stating that school efforts focused on promoting traditional forms of parental involvement fail to overcome difficulties and limitations faced by many families from Roma groups (Clifford and Humphries, 2018). Moreover, these school messages are likely to reinforce the families’ lack of ability to support children’s learning processes (Yamamoto and Sonnenschein, 2016) while undermining parents’ motivation to participate in and support their children’s education (Hoover-Dempsey et al., 2005; Hill et al., 2016). Literature on the acculturation models may help explain current and previous data. Researchers (e.g., Ho, 2014; Cote et al., 2015) argue that acculturation orientations influence parenting attitudes, beliefs, expectations, and, therefore, their endeavors to be involved in children’s school trajectories. The home-school cultural discontinuity requires parents from marginalized ethnic groups to adjust to novel cultural mores and norms for social interactions and unfamiliar rules and regulations (Berry et al., 2006; Motti-Stefanidi, 2019). However, the perceived life context (e.g., practical and logistic barriers, cultural values) and low cultural capital are likely to influence parental role constructions (e.g., self-efficacy, motivation) and parental involvement in education. Therefore, schools play a central role in shaping parental beliefs and identities regarding the involvement of parents from ethnic minority groups in education (Hill et al., 2016; Jeynes, 2018; Grace and Gerdes, 2019). As warned by Mapp et al. (2008), families need to perceive involvement in school as a part of their parental role and be sure of their ability to cope with these tasks. However, current findings suggest that educators may be missing opportunities to discuss with parents the inner workings of the educational system; and to expand and encourage parental practices to match their realities (e.g., parent–child communication about educational benefits, setting realistic academic expectations for their children, creating parent-teacher partnerships; Goodall and Montgomery, 2014; Clifford and Humphries, 2018). For instance, current evidence shows that school communication with families from Roma groups follows protocols distinct from those followed for mainstream families. In particular, participants reported that social assistants rather than teachers were likely to deliver information or set school-parent meetings. Furthermore, these meetings were primarily focused on children’s problems in school rather than on opportunities to help them grow and progress. As noted in prior literature (e.g., Bhopal and Myers, 2016; Hill et al., 2016), these approaches stressing dysfunctional behaviors may threaten school-family positive relationships and further hamper the effects of interventions to support families from marginalized ethnic groups in education.

Together, our findings add to the scarce literature by providing evidence against deficit model approaches portraying families with Roma backgrounds as lacking interest or willingness to support their children’s school life (e.g., Lauritzen and Nodeland, 2018; Parsons et al., 2018; Sime et al., 2018). Narratives reflect participants’ endeavors to engage in children’s educational trajectories while answering the school system’s demands. Data suggest acculturation attempts in public domains (i.e., accommodating mainstream expected behaviors) along with enculturation orientations regarding private domains (i.e., maintaining ethnic values and identity). Assuming acculturation as a process to boost changes in parenting beliefs, attitudes, and practices (Ho, 2014), current data raises questions on the effectiveness of the efforts undergone, either by the school or parents, and to analyze how school policies and actions are facilitating the integration of divergent cultural values and identities. For example, over the last decades, the differences emerging in data across rural and urban-living groups also divert attention from the political and economic investment underlying the acculturation processes of Roma groups. Despite having access to more resources (e.g., house-living conditions) and educational and health programs, living next to the mainstream systems, urban-living communities seem to show more indicators of separation strategies (e.g., preserving and communicating traditional pathways to children) than their peers from rural-living communities.

### 5.2. Section II. Participants’ engagement experience in the intervention program

The well-documented contribution of parental involvement to the positive educational process and achievement of students from marginalized groups (e.g., Flecha and Soler, 2013; Bhopal and Myers, 2016; Moreira et al., 2022) provides the impetus for school policies and efforts to foster positive home-school relationships. Consequently, many parents-and family-focused support programs were implemented inside and outside Portuguese borders (Nata and Cadima, 2019). However, anecdotal evidence often supports the low attendance rate and high dropout from parenting programs among participants from Roma groups. These data are similar to those of non-Roma low-resource and marginalized groups (see Shenderovich et al., 2019). In the current intervention, 76.5% of the parents invited on average attended 7.3 out of the eight intervention sessions. Of those, 10 participants (77%) completed all eight sessions, and 3 (33%) completed seven intervention sessions. This considerably high attendance level suggests that the intervention was feasible and acceptable to parents with a Roma background (Shenderovich et al., 2018).

As documented in previous studies (e.g., Hidalgo et al., 2016; Gonzalez et al., 2018; Giannotta et al., 2019), process-and content-related factors are highlighted as crucial ingredients to facilitate participants’ behavioral, emotional, and cognitive engagement in and with the sessions. For example, the facilitator’s personal characteristics (e.g., respect, warmth), domain, and cultural competence were crucial to favor trust-building relationships, decreasing initial resistance, and fostering engagement and overall responsiveness across the sessions. Notably, inviting parents as full partners in-person, promoting dialog across differences, and being welcoming and supportive were influential in promoting and sustaining parental involvement in sessions. Other essential components, such as increasing group cohesion and perceived content meaningfulness to participants’ personal life, may have contributed to participants’ interest, satisfaction, and outcomes (e.g., Koerting et al., 2013; Bamberger et al., 2014; Brager et al., 2021). In the current findings, participants pointed out that those features make them feel welcomed, valued, and supported, therefore increasing their openness to share concerns and overcome cultural barriers.

Furthermore, each participant was encouraged to collaborate in the sessions while suggesting ways to promote children’s school engagement. This strategy may have fostered parents’ sense of participatory partnership and encouraged session involvement. The literature argues that programs valuing parents’ existing knowledge and insights may foster their sense of self-efficacy and beliefs about how they can make a difference in supporting their children (Green et al., 2007; Nata and Cadima, 2019). As shown by the current data, the strategies and approaches used and the trustful relationships built were particularly important to counter acculturative hardships (e.g., discrimination, devaluing of cultural-based parental cognitions often adopted in parenting programs and training) and ensure adherence to the program. For example, the current findings indicate that urban-living participants with more experience in being enrolled in deficit-based interventions and actions (i.e., urban-living communities) showed more resistance to participating and engaging in the program. Moreover, data indicate that more exposure to programs and actions to strengthen home-school relationships did not translate into meaningful differences in parental role constructions and efforts compared to parents with even low access to resources (i.e., rural-living parents). The sample limitations underscore the need for cautious interpretations; however, this finding emphasizes the relevance of the delivery quality and the program’s characteristics, such as cultural adequacy and motivational enhancements (e.g., using a story-tool and goal-related activities). As supported in previous studies (e.g., Koerting et al., 2013; Holdsworth et al., 2014), those aspects were related to group and participant responsiveness by reducing the threat to participants’ self-efficacy. Moreover, the findings are in line with previous works (Hoover-Dempsey and Sandler, 1997; Shapiro et al., 2012; Barr and Saltmarsh, 2014; Ho, 2014; Ninković and Florić, 2018) pointing out the relevance of including a variety of strategies (e.g., gentle weekly reminders) to counter barriers threatening or hindering the implementation process.

There is some consensus in the literature that the extent to which participants enroll and engage in and with the intervention is related to overall intervention outcomes (Gross et al., 2003; Bamberger et al., 2014). Despite participants’ engagement in the sessions and the positive experiences reported, at the end of the program, no meaningful differences were found in the reported parental involvement activities. This finding is not surprising, given the complexity of the acculturation processes underlying parental involvement and the brief time frame for the intervention. As stated by Hill et al. (2016), to gain knowledge on how to support their children, families with ethnic minority backgrounds need to develop a shared identity, goals, and values with the school system. This acculturative process is acquired over the years through concerted and continued efforts of both schools and families (e.g., Jeynes, 2018; Kramer et al., 2021; Moreira et al., 2022). Moreover, while dynamic process changes are not at the hand of parents, teachers and school directors play a crucial role in shaping parental involvement patterns in children’s school life (e.g., Hoover-Dempsey et al., 2005; Jeynes, 2018; Parsons et al., 2018; Grace and Gerdes, 2019). Still, data show the participants’ willingness to understand better how to support their children and youth in school. Participants’ enrollment in this program without economic rewards (as usual in educational programs set for families with a Roma background) demonstrates families’ interest and willingness to participate in children’s education, as previously found in prior research on ethnic minority parents (e.g., Hill et al., 2016; Garcia and Guzman, 2020). Furthermore, participants’ statements provided evidence that enrollment in the sessions helped extend their parental role constructions underlying school participation, gaining insights into the inner-system workings of the educational system and expanding academic socialization efforts (e.g., parent–child communication about school). According to the literature, this tacit information is crucial for developing culturally-based parenting cognitions and efforts to participate in their children’s school life (Grace and Gerdes, 2019; Jeon et al., 2020). Most families from mainstream society hold this tacit information because they share attitudes, values, and identities with the school system. However, families from ethnic minority groups are less likely to have the cultural capital and competence to engage in traditional parental involvement (Clifford and Humphries, 2018; Parsons et al., 2018). For illustrative purposes, current findings evidenced that perspectives on parenting roles regarding education (e.g., the influential role parents may play in children’s academic and future life), which may be commonly held as a commodity by mainstream parents, were described by participants as a striking outcome of the program. Altogether, the current findings provide helpful insights into organizing resource-based efforts and building opportunities to sustain parental involvement, along with information on the most effective modes of transmission (Goodall and Montgomery, 2014; Henderson et al., 2020). We believe these findings will likely be helpful for school boards and educators willing to work with families from Roma groups. Moreover, despite preliminary, the indicators pointing to differences between rural versus urban living communities in acculturation processes may provide insightful information to rethink the effectiveness of fragmented actions and interventions aiming to “integrate” Roma groups without adequate monitoring assessment.

## 6. Limitations and future directions

Along with the valuable contributions of this study, some limitations must be acknowledged. For example, despite including participants from different Roma groups, our sample may not reflect the heterogeneity of Roma communities’ realities or even the distinct home-school relationship patterns across the country. Attending to the complexity of this phenomenon (i.e., acculturation and parental involvement) and the participants’ reported difficulties, the duration of the intervention may have been limited. A longer term of the program could have strengthened the impact of the intervention. Moreover, despite efforts to reduce language and understanding barriers, participants hold a limited verbal repertoire to express their perspectives and thoughts, which might have prevented capturing the nuances of the changes from pre-to post-interviews. Finally, the current study focused on mothers’ perspectives and relationships with education. Despite the central role played by mothers in children’s education, fathers are recognized by the participants and among the communities as figures of parental authority. Thus, future research may add relevant knowledge while addressing the perspectives, role constructions, and forms of involvement in children’s education of fathers with Roma backgrounds. Moreover, given the particular relevance of parents’ acculturation to understanding parental involvement in education, future research may consider including measures of parents’ acculturation orientations, acculturative stress, and sociocultural adjustment within rural and urban-living communities.

## 7. Conclusions and implications

Globally, the findings provide insights into how and the extent to which parents with a Roma background are involved in children’s education. Furthermore, despite the preliminary conclusions limited by the nonexperimental study design, data offer good insights into how schools can encourage parents’ engagement in and with school activities while upskilling parents and helping them mobilize to support their children and youth in the education process.

The efforts displayed by parents from Roma groups to participate in children’s education are often invisible to educators, and parents’ practices are portrayed as an essential source of children’s disengagement (Clifford and Humphries, 2018; Parsons et al., 2018). However, the current study provides evidence that participants with a Roma background are willing to engage in children’s education and value education as a tool for social mobility. Furthermore, parents have shown interest in improving their information and developing skills to support children in education. These findings challenge the prevailing deficit perspectives and may help school staff become aware of the forms of parental involvement used by Roma people and their willingness to participate in children’s education. This is particularly important because current findings suggest that school efforts to promote parental involvement are misaligned with the realities of families with a Roma background. Despite the initiatives to open avenues for communication, families still lack skills and information on the school system and on how to be involved in children’s education. This evidence may set the ground for a consensus among mainstream educators that parents from Roma groups play a passive role in children’s school life. Thus, despite accommodating expected behaviors, families still endorse cultural heritage values and identities diverging from mainstream-based values and expectations. The lack of tacit knowledge and cultural capital enlarges the cultural discontinuity underlying home-school relationships. Finally, findings emphasized the relevance of setting collaborative relationships to promote parents’ effective participation in children’s education. Authentic and trustful relationships built with and between participants favored engagement in the program, which *per se* should be considered a form of parental involvement in children’s education. Participants’ experiences and outcomes are expected to help expand policymakers, school administrators, and educators’ understanding and practices on how to reach families and build partnerships likely to promote the school success of children and youth with Roma backgrounds.

## Data availability statement

The datasets presented in this article are not readily available because data includes participants identifiable information. Requests to access the datasets should be directed to TM, taniateixeiramoreira@gmail.com.

## Ethics statement

The studies involving human participants were reviewed and approved by Scientific Ethics Committee of the University of Minho. The patients/participants provided their written informed consent to participate in this study.

## Author contributions

TM and PR conceived the original idea and supervised and coordinated the project and data collection. TM, PR, and EBL developed the theoretical framework and the design of the study. TM, JuM, CS, and JoM implemented the culturally sensitive program. CS, JuM, and JoM were involved in data collection and management. TM, JuM, and DM performed the data analysis and co-wrote the findings. CS, JoM, and DM reviewed the data analysis and findings. All authors contributed to the article and approved the submitted version.

## Funding

This study was conducted at Psychology Research Centre (UID/PSI/01662/2013), University of Minho, and supported by the Portuguese Foundation for Science and Technology and the Portuguese Ministry of Science, Technology and Higher Education through national funds (PTDC/PSI-GER/6847/2020), and co-financed by FEDER through COMPETE2020 under the PT2020 Partnership Agreement (POCI-01-0145-FEDER-007653). The TM was supported by a PhD fellowship from the Portuguese Foundation for Science and Technology (FCT) (SFRH/BD/125876/2016).

## Conflict of interest

The authors declare that the research was conducted in the absence of any commercial or financial relationships that could be construed as a potential conflict of interest.

## Publisher’s note

All claims expressed in this article are solely those of the authors and do not necessarily represent those of their affiliated organizations, or those of the publisher, the editors and the reviewers. Any product that may be evaluated in this article, or claim that may be made by its manufacturer, is not guaranteed or endorsed by the publisher.

## Footnotes

^1^According to the European Commission and Council of Europe (2012) definitions, the term, the term Roma is widely used in formal contexts to cover a wide diversity of groups (such as Roma, Sinti, Kale, Travellers, and the Eastern groups), including persons who identify themselves as Gypsies (the term used in Portugal). As an "umbrella" term, Roma recognizes the heterogeneity of lifestyles and cultural backgrounds and the need to be sensitive to framings that problematize the minority (Matras et al., 2015) and replace deprecatory terms (such as Gypsy) associated with a history of oppression. In the Portuguese context, people recognize themselves as Gypsies; however, as a political term of convenience, Roma is used to cover the heterogeneity among the different Gypsy groups in the Portuguese context.
