# Supplementary material for: Building partnerships in education through a story-tool based intervention: Parental involvement experiences among families with Roma backgrounds
Source: Front Psychol. 2023 Mar 9;14:1012568. doi: 10.3389/fpsyg.2023.1012568 (PMC10033949; doi:10.3389/fpsyg.2023.1012568)
Supplement: Supplementary file 1 [file Table_1.docx]

Supplementary Material

Table 1

*Frame story outline.*

| **Musca Journey Adventures**  The narrative comprises six chapters and depicts the story of Musca, an artic swallow, along her journey from the Artic to the Antarctic. Musca ran from the harsh cold of the arctic to meet summer on the other side of the Earth. While resting on a branch in Savannah, Musca shares their stories and adventures around the Earth with local birds and learns games and local stories from her new friends. The main narrative sets the stage for four shorter stories distributed across chapters.   \| **Chapter II**  ‘The rooster and the precious stone’ \| The first fable is about the Rooster and the precious stone. It is a short fable in which a Rooster is looking for food on a dunghill. While scratching, he comes across a particularly beautiful and brightening stone. Despite recognizing the value of the precious stone, the rooster keeps focused on his primary goal: finding food to feed himself. \| \| --- \| --- \| \| **Chapter III**  ‘The Donkey and the salt’ \| The second fable tells the story of a donkey used by his owner to transport salt from salt flats to home. During one of the journeys, the donkey fell accidentally into a stream, dissolving the salt. After the accident, the donkey load became lighter, and he enjoyed a less taxing journey home. On the following trip, the owner loaded the donkey with a cargo heavier than before, and the donkey, exhausted, fell by the wayside losing the salt. The owner was very upset and loaded the donkey with giant sponges on the next visit to the salt flats. When returning home loaded with salt, the donkey fell into a stream to set himself free of the cargo. Sill, and because of the sponges, the donkey almost died. Finally, the donkey was rescued by the owner. \| \| **Chapter IV**  “Zlateh The Goat” \| The third fable depicts the story of a family who decides they must sell their old goat to have money for their needs. Following this idea, the parents asked their oldest son to take the goat to the butcher. Along the journey, a storm arises, and they found themselves stuck in a hut. During the severe snowstorm, the boy and the goat took care of and benefited from one another, and the goat milk saved the boy. The family was distraught because they had disappeared during the snowstorm. When finally, both returned home, the family recognized the importance of the goat and saved their life. \| \| **Chapter VI**  “Wild and Young Animals of the Savannah” \| The fourth fable depicts the story of five wild young animals of the savannah who were feeling bored with their lives. These young animals decided to run away from their families and flee for an adventure in search of freedom in a territory populated only by young wild animals. Along their long journey, these animals realized that they had nothing to eat or drink. Their trip was not planned, and a few hours later they were very thirsty and hungry. Exhausted, they stopped to sleep and mysteriously disappeared during the night. No one ever saw them on the savannah. \| |
| --- | --- | --- | --- | --- | --- | --- | --- | --- |
