# Supplementary material for: Building partnerships in education through a story-tool based intervention: Parental involvement experiences among families with Roma backgrounds
Source: Front Psychol. 2023 Mar 9;14:1012568. doi: 10.3389/fpsyg.2023.1012568 (PMC10033949; doi:10.3389/fpsyg.2023.1012568)
Supplement: Supplementary file 2 [file Table_2.docx]

Supplementary Material

Table 2.

*Narrative and Sessions’ Overarching Goals.*

| *Narrative Chapter* | *Overarching Goals* | *Sessions* |
| --- | --- | --- |
| Chapter I: *The arrival*  Set the scenario and the main characters. | To elicit participants’ identification with story characters and openness to participate in the sessions and exchange knowledge. | Session I |
|  | ***Hands-on activity***: Crumpled Paper  To show how we can reshape our knowledge despite preserving our nature. |  |
| Chapter II – *Presentations*  Depicts the main characters’ characteristics, their life goals, and the relevance of these goals. The first fable is introduced in this chapter. | To explore their perspectives about the utility values of education, goals, expectations, and aspirations for their children’s future. Also, to elicit reflection on cultural meanings regarding parental roles towards supporting children in education and, broader, in life. | Session II |
| Chapter III – *The long journey*  Depicts the savannah bird reflections on the artic swallows’ long journeys worldwide. Also, this bird reflected on Musca’s efforts to approach goals and the strategies followed to pursue goals and counteract setbacks. | To instill reflection on the goal-guided trajectories and the implications of goal-setting generational gaps and generate culturally-adjusted strategies to pursue goals and manage acculturation hassles. | Sessions III and IV |
|  | ***Hands-on activity***: Patch of Plasticine  To elicit reflection on the role parents and family may play in shaping children’s trajectories, the barriers and consequences. |  |
| Chapter IV –*PLEA, the secret*  This chapter introduces the self-regulated learning cycle (SRL) as a tool to approach goals and tasks successfully. A new fable is presented to guide the readers through the SRL process and illustrate how it could be applied to different situations in life. | Building on their knowledge to equip parents with tacit knowledge on how to guide and support their children in their academic and life trajectories. Expand strategies and parental self-efficacy to participate in educational decisions. | Sessions V and VI |
|  | ***Hands-on activity***: The secret of my recipe  To apply PLEA while exchanging cultural information on traditional dishes. |  |
| Chapter V – *Proverb Game*  A game is outlined in this chapter. A set of proverbs and popular sayings are used to discuss concepts such as SRL, cooperation, and resilience. | To elicit discussion on topics related to SRL and open avenues to rebuild cultural meanings on parenting roles and future expectations and aspirations regarding education. | Session VII |
| Chapter VI – *Musca* *Farewell*  This chapter describes the moments preceding the goodbye of Musca. The savannah bird reflects on some lessons learned and presents a fable to Musca about the changes needed to adapt and cope with new environments. | To elicit discussion on cultural changes and their impact on ethnic identity, assess the potential cost-benefit of changes undergoing within the cultural groups, and negotiate changes to answer the participation in the mainstream systems. | Session VIII |
|  | ***Hands-on activity***: Origami  To prompt discussion on the ongoing construction of each individual and the contribution of microsystems (e.g., family, school) and cultural exchange. |  |
