# Supplementary figures and images for: Building partnerships in education through a story-tool based intervention: Parental involvement experiences among families with Roma backgrounds
Source: Front Psychol. 2023 Mar 9;14:1012568. doi: 10.3389/fpsyg.2023.1012568 (PMC10033949; doi:10.3389/fpsyg.2023.1012568)

## *Supplementary Material*

**Figure 1.**

*Discussion flow diagram.*

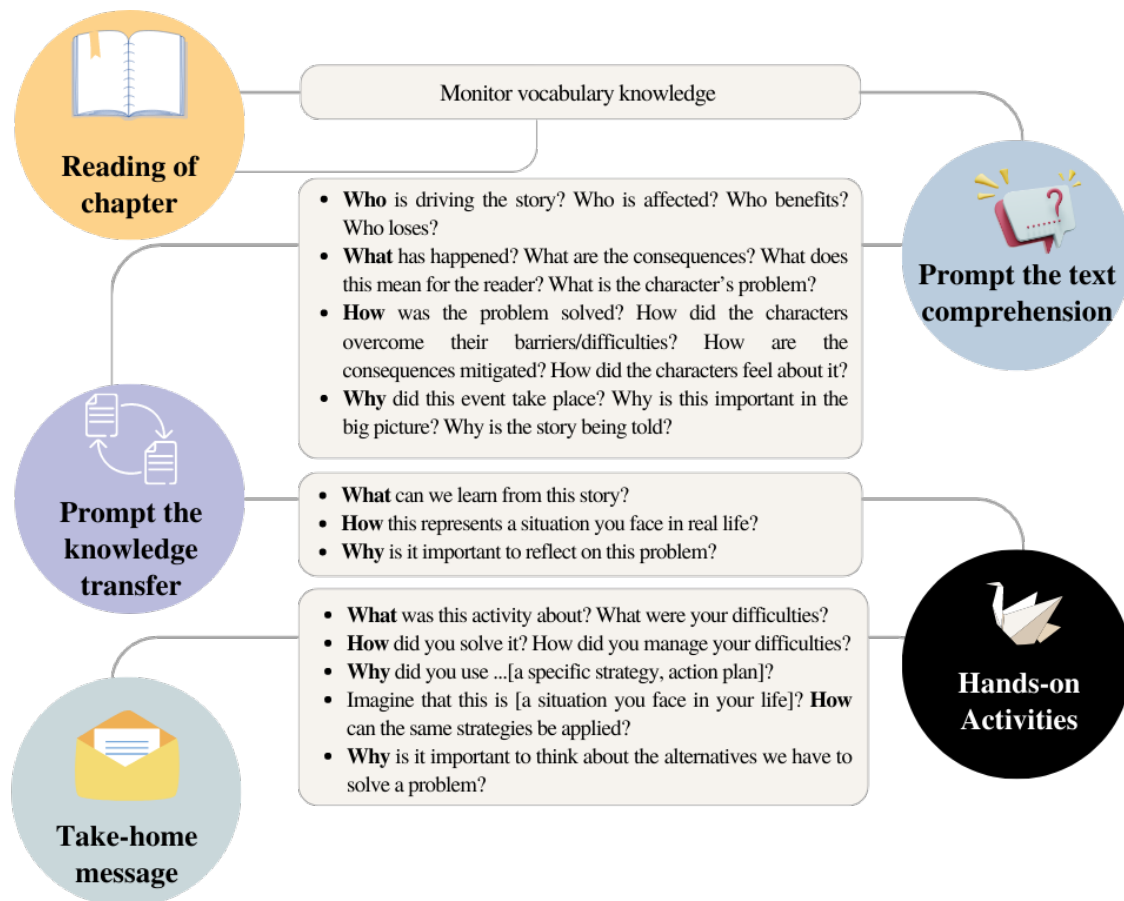

Supplement: Supplementary file 3 [file Image_1.pdf]
